# Untoward Effects of Cocaine

**Published:** 1887-05

**Authors:** 


### UNTOWARD EFFECTS OF COCAINE.

The varied and constant use of the hydrochlorate of cocaine by almost every practitioner of medicine renders imperative attention to the fact that this drug may act as a poison, since a very large number of well-authenticated cases of this character are now on record. While almost every reader of the current medical journals can recall the report of some case in which serious symptoms asserted themselves, the literature of the subject is so scattered that little attention has been given to it. Within the last few weeks, however, quite a large number of cases have been recorded in various medical periodicals, especially in Great Britain.

One of the first things which strikes the reader of these reports is the fact that no particular line of symptoms appears to be characteristic, and that the doses reported as producing toxic symptoms vary even more than the unlooked-for results themselves.

In England, where the practice of using cocaine hypodermatically for the purpose of producing local anæsthesia seems to be carried on much more fully than in this country, surgeons have reached the most varied conclusions as to the dose which may be safely given in this manner. Some insist that as much as four grains may be given under the skin, while others report very serious poisonings from the use of one grain. Thus Magill records the case of a strong, healthy man to whom he gave, previous to an operation for phimosis, a hypodermatic injection of one grain. In fifteen minutes the man was extremely pale and suffered from severe præcordial pain. The pulse was very slow and intermittent, but did not resemble that present when syncope is impending ; complete recovery took place in twenty minutes. More remarkable even than this is the record of seventeen cases gathered by Ziem, of Dantzig, in 1885, in which decided toxic effects occurred simply through instillations of the drug into the eye, and in which the amount of cocaine reaching the general system must have been very small indeed.

The symptoms which assert themselves are, as has just been stated, remarkable for their variety, and they range in severity from slight systemic depression to temporary blindness, or even syncope and collapse. Robson records a case in which aphasia followed its use in the removal of a nasal polypus, while Littlewood has seen great loquacity, and finally syncope, follow the hypodermatic administration of sixty minims of a six-per-cent. solution.

The interesting question in regard to this matter is the cause of the production of these symptoms and the best method for their avoidance. Unfortunately, there seems to be no fixed rule on which we can depend, since the causes themselves are as yet not by any means understood. Naturally idiosyncrasy occurs to the mind of every one as the most likely explanation, and this idea is somewhat strengthened, perhaps, by the various effects produced.

Again, it may be that the sudden and enormous demand has led to the placing on the market of adulterated or carelessly prepared samples of the drug, and if this be the case the reports of toxic effects should be constantly on the decrease, since at present the supply and demand are so nearly balanced. Whatever may be the explanation, it certainly seems of the greatest importance that the profession in general be reminded that cocaine is not to be used recklessly and without proper care.—*Medical News.*

